# Bridged Nucleic Acids Reloaded

**DOI:** 10.3390/molecules24122297

**Published:** 2019-06-21

**Authors:** Alfonso Soler-Bistué, Angeles Zorreguieta, Marcelo E. Tolmasky

**Affiliations:** 1 Instituto de Investigaciones Biotecnológicas Dr. Rodolfo A. Ugalde, Instituto Tecnológico de Chascomús, CONICET, Universidad Nacional de San Martín, San Martín 1650, Argentina; asoler@iib.unsam.edu.ar; 2 Fundación Instituto Leloir, IIBBA-CONICET, Buenos Aires C1405BWE, Argentina; azorreguieta@leloir.org.ar; 3 Center for Applied Biotechnology Studies, Department of Biological Science, California State University Fullerton, Fullerton, CA 92834-6850, USA

**Keywords:** oligonucleotides, bridged nucleic acids, locked nucleic acids, antisense, antibiotic resistance, hypercholesterolemia, myotonic dystrophy, CRISPR, Cas9, hematologic malignancies

## Abstract

Oligonucleotides are key compounds widely used for research, diagnostics, and therapeutics. The rapid increase in oligonucleotide-based applications, together with the progress in nucleic acids research, has led to the design of nucleotide analogs that, when part of these oligomers, enhance their efficiency, bioavailability, or stability. One of the most useful nucleotide analogs is the first-generation bridged nucleic acids (BNA), also known as locked nucleic acids (LNA), which were used in combination with ribonucleotides, deoxyribonucleotides, or other analogs to construct oligomers with diverse applications. However, there is still room to improve their efficiency, bioavailability, stability, and, importantly, toxicity. A second-generation BNA, BNA^NC^ (2′-*O*,4′-aminoethylene bridged nucleic acid), has been recently made available. Oligomers containing these analogs not only showed less toxicity when compared to LNA-containing compounds but, in some cases, also exhibited higher specificity. Although there are still few applications where BNA^NC^-containing compounds have been researched, the promising results warrant more effort in incorporating these analogs for other applications. Furthermore, newer BNA compounds will be introduced in the near future, offering great hope to oligonucleotide-based fields of research and applications.

## 1. Oligonucleotides and Analogs

**Figure 1:**
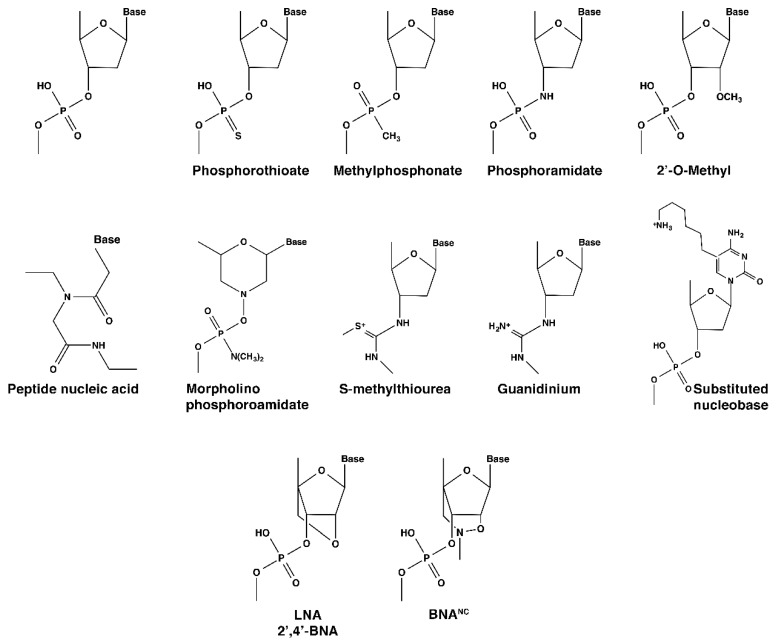
Chemical structures of nucleotide analogs.

Oligonucleotides are short oligomers composed of ribonucleotides or deoxyribonucleotides. They have multiple uses in basic research, diagnostics, and therapeutics. Their most basic and widespread use is in primer-based techniques, which are used in the most diverse kinds of biological research or development projects, such as polymerase chain reaction (PCR), library construction, single nucleotide polymorphisms detection, gene silencing, tiling arrays, and many others. Besides these applications, oligonucleotides were also found to be immensely useful in the development of gene-silencing techniques. Several approaches were attempted to reduce the undesirable expression of genes, utilizing a variety of strategies, the vast majority of which have in common the utilization of antisense oligonucleotides [1,2,3]. Many of these compounds are known with different names descriptive of their mechanism of action, like external guide sequences [4,5], ribozymes [6,7], aptamers [8,9], short interfering RNA [10,11,12], and microRNA [13,14]. These compounds interfere with gene expression by steric hindrance of transcription or translation, or by inducing enzymatic cleavage of the target mRNA [3,4,11,13]. The rapid increase in the techniques and applications for which these compounds are integral components, together with the progress in nucleic acid research, led to the design of analogs that are more appropriate to enhance efficiency and achieve specific objectives in each case. In general, nucleic acid analogs should a) have higher affinity per nucleotide unit than the cognate sequence, without changing the structure of duplexes, b) be resistant to nucleases, and c) have low toxicity. The first oligonucleotide analogs consisted of relatively minor modifications to the natural counterparts, such as the replacement of an oxygen atom by sulfur (phosphorothioate) [15,16], methyl (methylphosphonate) [17], or amino groups (phosphoramidate) (Figure 1). The value of using nucleotide analogs was illustrated by the first FDA approved antisense drug, fomivirsen, a 21-nucleotide oligomer composed of phosphorothioate units designed for the treatment of cytomegalovirus retinitis [10,18]. Subsequently, more modifications were introduced to the nucleotide molecule, such as adding chemical groups to the 2′ position of the ribose as in 2′-*O*-methyl compounds (Figure 1) or making more drastic structural changes replacing or modifying the ribose, substituting the nature of the bonds, or modifying the charge of the oligonucleotide, obtaining neutral or cationic derivatives. Examples of these compounds are morpholino phosphoroamidates [19], peptide nucleic acids [20,21], nucleotides with modified nucleobases [22], guanidinium-linked oligomers [23], and locked nucleic acids (LNA) [24] (Figure 1). Exhaustive listings and a description of oligonucleotide analogs and their applications can be found in recent reviews [3,6,16,23,25,26,27]. A derivative of LNA, 2′-*O*,4′-aminoethylene bridged nucleic acid (BNA), also known as 2′,4′-BNA^NC^ (BNA^NC^) [28,29,30,31] (Figure 1) has been recently introduced and other derivatives have followed or are in development. As a consequence, LNA is considered the earliest generation of bridged nucleic acids (BNA). This review will focus on the properties and applications of BNA^NC^-containing compounds.

## 2. LNA—A Brief Overview

LNA compounds, first introduced in the late 1990s, are bicyclic nucleotide analogs in which the furanose ring is modified by the introduction of a methylene group linking the 2′-oxygen and the 4′-carbon (2′-*O*,4′-methylene-β-d-ribofuranosyl nucleotides) [32,33,34,35,36,37]. They are characterized by reduced flexibility of the ribose residue and exist in a locked *N*-type conformation, which favors the formation of stable duplexes with DNA or RNA [34]. Numerous structural and thermal stability studies on complexes formed by LNA oligomers and complementary DNA or RNA oligonucleotides showed higher melting temperatures and specificity when compared to the unmodified isosequential compounds [32,34,37,38,39]. LNA-containing oligomers are usually synthesized as chimeras containing a combination of ribonucleotide/deoxyribonucleotide or other nucleotide analogs and LNA residues [40,41,42,43,44]. The incorporation of LNA in oligonucleotides leads to melting temperature increases of 3–9 °C per residue [45]. Most LNA-containing chimeras can be classified into two kinds, gapmers and mixmers. Gapmers consist of oligomers where the LNA residues are located at the ends of the compound. Mixmers include the LNA and the other residues interspersed in different configurations throughout the sequence. LNA-containing oligomers have been used for diagnostics and other applications, as probes or primers for hybridization, amplification, mutagenesis, sequencing, and SNP genotyping [46,47,48,49,50,51,52,53], in addition to gene repair [54] and antisense drugs [13,41,42,55]. While the increased binding capacity is advantageous in many antisense applications, it can also be detrimental due to the formation of duplex structures that cannot be recognized as substrates by the enzymes recruited to degrade the target molecule. The high affinity of LNA-containing oligonucleotides can also result in toxic effects due to unspecific off-target binding. Fortunately, recent studies showed that LNA-containing oligomers were innocuous in primates [56] and relatively safe in humans [57,58,59]. Furthermore, these compounds failed to show genotoxicity [60]. However, these results are far from definitive; other studies showed hepatoxicity [61,62,63,64]. Although the toxicity determinations of LNA-containing oligomers are encouraging, case-by-case studies will decide the possibility of development as therapies for human disease. At the moment, numerous potential drugs based on this nucleic acid analog are already in clinical trials (e.g., Miravirsen, MRG-106, and ISTH0036) [10,11]. An obstacle in the development of LNA-containing oligomers as drugs, particularly in silencing prokaryotic genes, is the low or null uptake by bacterial cells. Gymnotic uptake of LNA-containing oligomers was shown in eukaryotic and prokaryotic cells [65,66,67]. However, although internalization by diverse pathways and reasonable levels of activity were reported in the case of eukaryotic cells [68,69,70,71,72], the levels of internalization into bacterial cells seems not to be enough for productive inhibition of gene expression [66]. LNA-containing oligomers could be delivered to cultured eukaryotic cells by transfection reagents [73,74,75,76]. Other strategies to facilitate uptake, such as conjugation to cell penetrating peptides (CPP), which are usually cationic, has been challenging and reports of their utilization to silence gene expression are scarce. Turner et al. [77] reported the attachment of a CPP to an LNA/2′-*O*-methyl oligonucleotide; cell uptake of this compound was significantly increased with respect to the naked antisense, but levels of inhibition of gene expression were disappointing. An LNA/DNA gapmer designed to target the amikacin resistance *aac(6*′*)-Ib* gene mRNA and elicit cleavage by the endogenous RNase P [4] was covalently bound to a CPP. The compound produced a modest reduction in the levels of resistance to amikacin in a clinical *Acinetobacter baumannii* isolate [78]. LNA-containing compounds were also delivered inside target cells using nanoparticles [79]. An 8-nucleotide LNA oligomer complementary to the oncogenic miR21 included in micelles could be delivered inside cancer cells and induced apoptosis [79]. Furthermore, these assays showed tumor growth inhibition in an animal model [79].

In summary, LNA-containing compounds show great promise as therapeutic agents [42,80,81]. However, more research is needed to improve cell penetration while keeping their biological activity and reduced toxicity. Comprehensive descriptions of properties and applications of LNA containing oligomers have been recently published [82,83,84,85,86].

## 3. BNA^NC^

The success and advantages of LNA-containing oligomers for diverse applications stimulated the search for similar compounds, improving their properties. Many derivatives were recently introduced, like BNA^NC^ with different substitutions at the N atom (of which a methyl group is the most commonly used to date) (Figure 1) [87], 2′-*O*,4′-*C*-ethylene-bridged nucleic acid (ENA) [88], 2′-*O*,4′-*C*-methylenoxymethylene-bridged nucleic acid [89], or unlocked nucleic acids (UNA) [90,91]. The bridge in the different BNA compounds can have a different number of members in the ring, the most widely used to date being BNA^NC^, a six-member ring [30,31]. Oligonucleotides containing BNA^NC^ are more resistant to nucleases and less toxic than isosequential compounds containing LNA residues, they show high thermal stability and water solubility, and elicit RNase H degradation of a target RNA [29,62,87,92].

Twelve-mer oligodeoxynucleotides containing a variable number of BNA^NC^ or LNA residues showed a similar increase in melting temperature (Tm) per modified residue (4.7 to 7.0 °C) in comparisons of duplex stabilities with complementary single-stranded RNA (ssRNA). However, this was not the case for duplexes with complementary single-stranded DNA (ssDNA) [29]. Increasing the number of BNA^NC^ residues in the oligonucleotides did not result in a significant Tm increase (−1.0 to 1.8 °C) as was the case when the analog used was LNA (1.3 to 3.0 °C) [29]. While the Tm values of the unsubstituted oligodeoxynucleotide in duplex with complementary ssRNA was about 5 °C higher than with complementary ssRNA, the Tm values for substituted oligonucleotides were higher for duplexes with complementary ssRNA. Furthermore, the values were higher for oligonucleotides substituted with BNA^NC^ than those that included LNA residues [29]. These results indicate that BNA^NC^ residues conferred a higher RNA selective binding affinity to the antisense oligomers. BNA^NC^-containing oligonucleotides also showed higher mismatch discrimination properties as well as more stable triplexes than LNA-containing oligomers [29,93]. BNA^NC^-containing oligonucleotides have the potential to be utilized in diverse applications. In this review, we will focus on their utilization for diverse uses. The different applications of the compounds reviewed in this article are summarized in Table 1.

### 3.1. Antisense Inhibition of Resistance to Amikacin by a BNA^NC^-Containing Oligomer

The inhibition of the expression of genes coding for antimicrobial resistance enzymes by diverse antisense mechanisms is the object of intense investigation, as a way to deal with the growing multiresistance problem [4,98,99,100,101]. An active antisense molecule could be combined as an adjuvant to the cognate antibiotic to treat resistant infections. The concept of treating resistant infections with combinations of antibiotic/inhibitor of resistance has reached the stage of human use in the case of β-lactam antibiotics that are administered in combination with β-lactamase inhibitors [99,102,103]. However, combinations of other kinds of antibiotics with inhibitors of resistance are still in experimental stages [99,104]. In particular, antisense inhibition of antibiotic resistance genes was explored using oligomers of different natures that interfere with the expression of resistance by different mechanisms [41,42,78,98,101,105,106,107,108]. The *aac(6*′*)-Ib* gene codes for an acetyltransferase responsible for the resistance to amikacin and other aminoglycosides found in the vast majority of AAC(6′)-I-producing Gram-negative clinical isolates [104,109]. An antisense oligodeoxynucleotide complementary to a duplicated sequence located at the translation initiation location of the *aac(6*′*)-Ib* allele found in a clinical *Acinetobacter baumannii* isolate [110] inhibited translation in vitro [94]. An isosequential 15-residue antisense mixmer, including four BNA^NC^ and 11 deoxynucleotide residues, was covalently bound to the permeabilizing peptide (RXR)_4_XB (R, arginine; X, 6-aminohexanoic acid; B, β-alanine) to generate a compound resistant to nucleases and capable of penetrating the Gram-negative envelope to reach the cytosol. This BNA^NC^-containing mixmer, designated CPPBD4, successfully inhibited growth in a liquid culture containing amikacin. Furthermore, a combination of CPPBD4/amikacin reduced the mortality of *Galleria mellonella* infected with amikacin-resistant *A. baumannii* to levels comparable to those of the uninfected controls [94].

BNA^NC^-containing oligomers were also researched as elicitors of RNase P-mediated specific mRNA degradation, an antisense methodology known as external guide sequence (EGS) technology [4,111]. In this approach, antisense oligomers, known as EGSs, interact with the target mRNA, forming a structure that is recognized as a substrate by the endogenous RNase P. Then, the enzyme cleaves the mRNA, preventing its translation [5]. Early experiments showed that EGS molecules efficiently reduced the expression of *aac(6*′*)-Ib* and levels of resistance to amikacin [108]. Analysis of various nuclease-resistant oligonucleotide analogs indicated that DNA/LNA hybrid oligomers were efficient EGSs that reversed *aac(6*′*)-Ib-*mediated resistance to amikacin in a hyperpermeable *Escherichia coli* strain [42]. Furthermore, conjugation of a selected DNA/LNA hybrid oligomer to the cell permeabilizing peptide (RXR)_4_XB reduced the levels of resistance to amikacin of an *aac(6*′*)-Ib-*containing *Acinetobacter baumannii* clinical isolate [41,78]. Assessment of isosequential hybrid oligomers containing BNA^NC^ in the place of the LNA residues showed that they failed to elicit cleavage of the mRNA at levels comparable to those found when testing LNA/DNAs [41].

### 3.2. Reversion of Splicing and Reduction of RNA Foci in Myotonic Dystrophy Cells by BNA^NC^ Gapmers

Myotonic dystrophy type 1 patients may suffer from skeletal muscle weakening and wasting, abnormalities in heart function, cataracts, breathing problems, speech and swallowing disorders, and other impairing symptoms [112]. The molecular basis of this condition is the presence of a CUG repeat expansion within the *DMPK* gene 3′-untranslated region [113,114]. This kind of genetic modification, in which a group of nucleotides that in healthy genes is repeated a variable number of times, exists in an abnormally high number of repeat-characterized diseases, known as microsatellite expansion disorders [115,116]. The *DMPK* gene anomaly causes the mRNA to remain in the nucleus and to form foci structures, resulting in defects in developmentally regulated alternative splicing [117]. Removal of the toxic RNA species using antisense oligomers that induce RNase H cleavage is being intensely researched as a therapeutic strategy. Early work by researchers at IONIS Pharmaceuticals utilizing a gapmer consisting of a short DNA stretch flanked by 2′-*O*-methoxyethyl residues designed to induce cleavage of the toxic RNA in muscle cells showed encouraging splicing changes, but stopped short of the goals of the trial [95]. The potency of these compounds can be substantially increased by replacing the 2′-*O*-methoxyethyl analogs with LNA residues [62]. However, antisense LNA-containing gapmers showed high hepatotoxicity [62,63,64]. The potency and toxicity of BNA^NC^-containing gapmers as compared to isosequential LNA-containing gapmers was recently determined [95]. Both compounds were nearly equally efficient in inducing the cleavage of the CUG repeat expansion-containing mRNA in cells transformed with a plasmid designed to express this RNA species. However, comparison of the toxicity of both antisense compounds showed that the LNA-containing gapmer induced an increase in caspases, the apoptosis effector proteins, that was not observed with BNA^NC^-containing gapmers [95]. Interestingly, a comparison of the region targeted showed that a gapmer complementary to a non-repetitive region of the RNA was more specific in eliciting degradation of the toxic RNA than a gapmer complementary to a segment containing the CUG repeats (Table 1) [95]. These studies indicate that the use of BNA^NC^-containing gapmers could open new venues for developing therapies against myotonic dystrophy type 1.

### 3.3. Reduction of Cholesterol Levels by BNA^NC^ Mixmers in Hypercholesterolemic Mice

Proprotein convertase subtilisin/kexin type 9 (PCSK9) plays a role in the maintenance of cholesterol balance [118]. Gain-of-function mutations in this gene are associated with an increase in low-density lipoprotein cholesterol levels (i.e., autosomal dominant hypercholesterolemia), a known risk factor for coronary heart disease [119]. Conversely, loss-of-function mutations are responsible for low plasma low-density lipoprotein cholesterol (LDL-C) levels and reduced incidence of cardiovascular disease [120]. These phenotypes are the consequence of the ability of PCSK9 to interact with the LDL receptor (LDLR). The complex PCSK9–LDLR is transported from the cell’s surface to the endosome, where LDLR is degraded [121]. The involvement of PCSK9 in the modulation of LDL-C levels led to attempts to suppress its synthesis or activity that resulted in the development of drugs that have been approved by the FDA [122,123,124,125,126]. In particular, the inhibition of the expression of PCSK9 by antisense oligonucleotide analogs was attempted by several research groups. A 2′-*O*-methoxyethyl-modified phosphorothioate oligonucleotide analog modestly reduced hepatic PCSK9 mRNA and LDL-C in treated mice [127]. In another study, an LNA-containing gapmer was found to be more efficient at lowering PCSK9 mRNA and LDL-C while increasing LDLR levels [74]. However, LNA-containing gapmers usually show high hepatotoxicity. BNA^NC^-containing oligomers could be a better option to design compounds that show high efficiency without the high levels of toxicity observed when the analog used is LNA. Yamamoto et al. [92] tested LNA- and BNA^NC^-containing antisense mixmers in cultured cells as well as in mice. Cells transfected with mixmers containing one or the other analog showed a dose-dependent reduction of PCSK9 mRNA and increase of LDLR protein levels. Also, the administration of these compounds to atherogenic diet-fed mice biweekly for six weeks resulted in a reduction of PCSK9 mRNA and LDL-C as well as an increase in high-density lipoprotein cholesterol. However, the comparison also showed that the animals treated with the BNA^NC^-containing antisense mixmer experienced a reduction of LDL-C after a shorter time and tolerance was higher. These comparative studies identified BNA^NC^-containing antisense compounds as better candidates than LNA-containing compounds.

### 3.4. Diagnostics of Hematologic Malignancies by the Detection of Somatic Mutations Using BNA^NC^ Mixmers

DNA methylation, an epigenetic modification, is involved in critical cellular processes, such as replication and transcription. Hypomethylation is correlated to some human cancers [128]. The human DNA methyltransferase 3A (DNMT3A) is a 130 kDa protein that includes three domains, one of which is the *S*-adenosyl methionine-dependent methyltransferase that recognizes and binds DNA to catalyze the transfer of a methyl group to the C5 position of cytosine from *S*-adenosyl methionine [129]. The nuclear DNMT3A molecules can exist in oligomeric form as dimers, tetramers, and larger structures held by interactions of binding interfaces in the methyltransferase domain [130].

Numerous hematologic malignancies are characterized by the presence of somatic mutations in the DNA methyltransferase 3A (DNMT3A) gene [128]. Mutations on this gene are found in up to 35% of cases of different myeloid malignancies [131,132,133,134,135,136,137]. The detection of mutations in this gene could, therefore, be a component of a group of tests to predict a higher risk of myeloid malignancies [138,139]. Techniques used to detect DNMT3A mutations include DNA sequencing, high-resolution DNA melting, restriction fragment length polymorphism, and denaturing high-performance liquid chromatography [140,141,142,143,144]. A microsphere-based suspension assay that utilizes oligonucleotide analogs, LNA- or BNA^NC^-containing, as probes specific for wild type or mutant alleles was more efficient than direct sequencing [96]. In this study, LNA- or BNA^NC^-containing oligonucleotides specific for the wild type or four mutations within the codon R882 were coupled to fluorescently labeled microspheres, which were used in hybridization assays against DNMT3A amplicons. The utilization of LNA- or BNA^NC^-containing analogs facilitated the design of sequences that can discriminate between sequences that differed in a single nucleotide. A comparison of isosequential oligonucleotides that differ in the nature of the analog residues showed that BNA^NC^-containing compounds showed the highest sensitivity, as well as specificity. These researchers concluded that BNA^NC^-containing probes, coupled with fluorescently labeled microspheres, are suitable reagents to detect DNMT3A R888 mutations [96].

### 3.5. Incorporation of BNA^NC^ Residues to crRNA as an Enhancer of Cas9 Endonuclease Specificity

**Figure 2:**
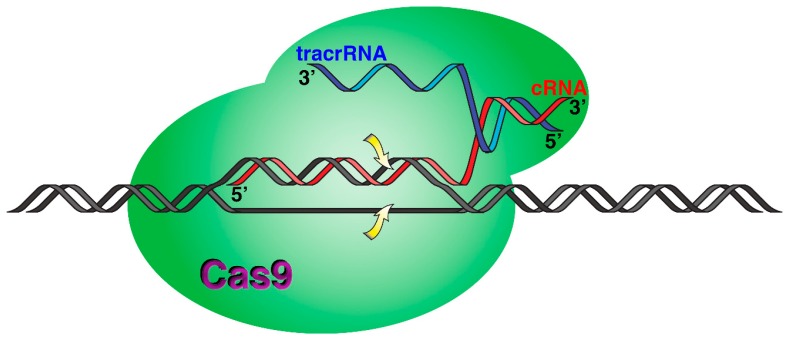
Molecular mechanism of Cas9-mediated digestion of a target DNA. The crRNA and tracrRNA are shown in red and blue, respectively. The arrows indicate the point of cleavage on each target DNA strand.

Bacteria have evolved numerous strategies to defend against the presence of foreign genetic material, such as bacteriophage genomes, transposons, and plasmids. They include restriction-modification systems, abortive infections and adsorption blockage, and surface exclusion [145,146,147]. While these systems are relatively unspecific, the latest discovered defense system, known as “clustered regularly interspaced short palindromic repeats” (CRISPR) is specific and, in a surprisingly analogous mode to vertebrate immune systems, it requires previous exposure to the foreign genetic material to create a memory record that can elicit a quick response in future exposure events [148]. In this system, small guide RNA molecules (crRNAs) guide the sequence-specific cleavage of foreign nucleic acids [149]. However, differences in molecular mechanisms permitted the classification in two classes, class 1 and 2, which depend on a multiprotein complex or a single protein, respectively [149]. Each class is further divided into three types. In particular, the class 2, type II system, which depends on the endonuclease Cas9 (Figure 2), has been developed as a tool for numerous applications, such as the generation of mutants, gene editing, bacterial species identification and typing, antibacterial agents, genome-wide screening in mammalian cells, regulation of gene expression (through the use of dCas9, a derivative that lost the cleavage activity), and others [148,149,150,151,152].

The Cas9 system relies on two RNA molecules, a crRNA that includes a 20-nucleotide sequence complementary to the target DNA and a trans-acting molecule (tracrRNA) that acts as bridge between the crRNA and the Cas9 endonuclease (Figure 2) [153,154,155]. Experiments in which the tracrRNA and crRNA are covalently bound, forming a single guide RNA (sgRNA) showed that sgRNAs form viable complexes that result in target cleavage [155]. The Cas9 protein first recognizes protospacer-adjacent motif (PAM) sequences on the target DNA. The role of the PAM sequences is to help in distinguishing self from foreign DNA. Then, a crRNA 20-nucleotides region pairs with the target DNA, forming the multicomponent complex that causes Cas9 double-stranded blunt-ended cleavage [153,156,157]. The Cas9 system has been widely adapted for gene editing and other applications [152,158,159,160,161]. An important consideration in the use of CRISPR-Cas9 as a tool is its specificity. While mutations in the PAM sequences usually interfere with cleavage of the target, mutations within the target sequence that interacts with crRNA (or sgRNA) can be tolerated, permitting digestion at off-target locations [162,163]. Since enhancing the specificity of cleavage is such an important aspect for numerous applications of CRISPR-Cas9, it is not surprising that several approaches have been tested to reduce off-target action [97,164,165,166]. The vast majority of these studies focused on the Cas9 protein [165,167,168,169,170]. Few others concentrated on modifications to the crRNA [97,170,171,172]. Cromwell et al. recently took advantage of the enhanced mismatch discrimination of BNA^NC^-containing oligomers to design crRNA molecules that elicit cleavage specificity by the cas9 endonuclease [97]. Two crRNA molecules directed to the Wiskott–Aldrich syndrome (WAS) [173] and the homeobox EMX1 [174] genes that are known to also affect off-target sites were used to test the effect of replacing from one to four nucleotides with BNA^NC^ residues. In vitro cleavage assays were carried, out using as the target the wild type WAS or EMX1 sequences and five sequences including one, two, or three nucleotide substitutions for each of the genes. These wild type and modified target sequences were incubated in the presence of the endonuclease and crRNA or the isosequential variants, including one to eight BNA^NC^ residue replacements. While the crRNA molecules directed cleavage of the wild type and all of the modified targets, a crRNA containing BNA^NC^ residues at positions 12–14 isosequential to the EXM1 crRNA (Table 1) specifically guided cleavage of the wild type target. Similarly, a crRNA containing BNA^NC^ residues at positions 10–12 (Table 1) guided cleavage of the wild type and only one of the modified targets. These results showed a significant enhancement of specificity in vitro when three nucleotides were replaced by BNA^NC^ residues. Interestingly, BNA^NC^-containing crRNA molecules are compatible with Cas9 variants with improved specificity. Following these encouraging results, experiments to find out if the enhanced specificity observed in the in vitro experiments described above also occurs in vivo were carried out. Cas9-producing U2OS and HeLa cells were transfected with the unmodified and modified crRNA molecules, targeting the WAS and EXM1 genes. The levels of cleavage at the target and off-target sites were consistent with those observed in vitro. This work established that BNA^NC^-containing crRNAs could be a venue for improving Cas9 cleavage specificity [97].

## 4. Final Remarks

In the past few years, oligonucleotides have been used for an increasing number of applications in basic science, as well as in clinics and other settings. In particular, applications related to human health include detection and diagnostics of pathologies caused by mutations, silencing of undesirable genes responsible for numerous genetic conditions, or interference with the expression of bacterial or viral genes to fight infection. The increased utilization of these compounds requires the constant search for improvement of their properties. The key aspects that enhance the usability and efficiency of oligonucleotides are their stability, specificity, and bioavailability. While the latter has been dealt with by conjugating the compounds to permeabilizing peptides, using transfecting agents, packaging them into liposomes, and combining them with nanoparticles, stability and specificity were dramatically improved by the substitution of ribonucleotide or deoxyribonucleotide residues with analogs. The success observed by these chemical modifications incentivized the search for new and varying nucleotide analogs that enhanced the resistance to nucleases, specificity, affinity, and activity of the oligomers. The first-generation BNA, known as LNA, has been used for several applications with great success. However, despite the promising results achieved with LNA-containing oligomers, toxicity has been an impediment for their development as therapeutic agents. Conversely, oligonucleotide analogs, including the second generation of BNA compounds, BNA^NC^, show lower toxicity while preserving and, in some cases, improving on the LNA-containing oligomers. Furthermore, next-generation BNA compounds are in the pipeline, and some are or will soon be available for experimentation [87,175]. The success experienced using these nucleotide analogs, and the vigorous efforts to continue developing next-generation variants offer promising alternatives to continue the development of new and improved applications of oligonucleotides for research as well as diagnostics and therapeutics.

## Figures and Tables

**Table 1:** BNA^NC^ applications.

Organism	Function or Disease	Target	Chemical Nature of Oligonucleotide	Sequence of Active Oligomer	Reference
*A. baumannii*	Resistance to aminoglycosides	*aac(6′)-Ib*	BNA^NC^/DNA conjugated to (RXR)_4_XB ^1^	(RXR)_4_XB-Cys-SMCC-C6 amino-cTgctGcgtAacaTc	[94]
Cell lines	Myotonic dystrophy type 1	*DMPK* ^2^	BNA^NC^/DNA gapmer	CGGAGcggttgtgaaCTGGC	[95]
Murine, human cell lines, and mice	Hypercholesterolemia	*PCSK9* ^3^	BNA^NC^/DNA mixmer	CCaggCCTaTgagggTgCCg	[92]
Human gene	Hematologic malignancies	*DNTM3A* ^4^	BNA^NC^/DNA mixmers	cgccaAgcgGctcatgtt cgccAAgcagctcAtgtt cgccAAgtgGctcAtgtt cgccaAggggCtcatgtt cgccAAgctgCtcAtgtt	[96]
Human gene	CRISPR-Cas9 specificity	*WAS* ^5^	crRNA with BNA^NC^ substitutions	uggauggagGAAugaggagu	[97]
Human gene	CRISPR-Cas9 specificity	*EXM1* ^6^	crRNA with BNA^NC^ substitutions	gaguccgagcaGAAgaagaa	[97]

^1^ R, arginine; X, 6-aminohexanoic acid; B, β-alanine; ^2^ Dystrophia myotonica protein kinase; ^3^ Proprotein convertase subtilisin/kexin type 9; ^4^ Human DNA methyltransferase 3A; ^5^ Gene responsible for Wiskott-Aldrich Syndrome; ^6^ Homeobox protein EMX1.
